# Cyclic di-GMP regulates bacterial colonization and further biocontrol efficacy of *Bacillus velezensis* against apple ring rot disease *via* its potential receptor YdaK

**DOI:** 10.3389/fmicb.2022.1034168

**Published:** 2022-12-16

**Authors:** Huiling Gong, Wenxiao Jiang, Yang Yang, Yue Zhang, Xufei Chen, Wei Li, Panlei Yang, Zhenshuo Wang, Qi Wang, Yan Li

**Affiliations:** ^1^Department of Plant Pathology, College of Plant Protection, China Agricultural University, Beijing, China; ^2^Chongqing Key Laboratory of Plant Disease Biology, College of Plant Protection, Southwest University, Chongqing, China

**Keywords:** *Bacillus velezensis*, c-di-GMP, biofilm, colonization, biological control, apple ring rot disease

## Abstract

*Bacillus* species are among the most investigated beneficial bacteria and widely used in agricultural systems as biological control agents. Its biocontrol efficacy is controlled by diverse regulators. Cyclic diguanylate (c-di-GMP) is a nearly universal second messenger in bacteria and modulates various important physiological processes, including motility, biofilm formation, antifungal antibiotic production and host colonization. However, the impact of c-di-GMP on biocontrol efficacy of beneficial bacteria is unknown. *Bacillus velezensis* PG12 is an effective biocontrol strain against apple ring rot disease caused by *Botryosphaeria dothidea*. In this study, the contribution of c-di-GMP to biocontrol efficacy of *B. velezensis* PG12 was investigated. Deletion of single gene encoding diguanylate cyclase or phosphodiesterase did not affect its biocontrol efficacy against apple ring rot. However, artificial modulation of c-di-GMP level in the cells leads to a significant change of biocontrol efficacy, suggesting that c-di-GMP positively regulates biocontrol efficacy of *B. velezensis* PG12 against apple ring rot disease. More evidences indicate that c-di-GMP does not affect the antagonistic activity of *B. velezensis* PG12 against *B. dothidea in vitro* and *in vivo*, but positively regulates biofilm formation of *B. velezensis* PG12 and its colonization on apple fruits. Importantly, deletion of *ydaK* could rescue the inhibition of biofilm formation, bacterial colonization and biocontrol efficacy caused by low c-di-GMP level, indicating that YdaK is the potential c-di-GMP receptor to regulate biofilm formation, colonization and effective biological control. However, YdaK did not affect the antagonistic activity of *B. velezensis* PG12 against *B. dothidea*. Based on these findings, we propose that c-di-GMP regulates biofilm formation, subsequently the bacterial colonization on apple fruits and thus biocontrol efficacy of *B. velezensis* through its receptor YdaK. This is the first report showing that c-di-GMP plays a role in biocontrol efficacy of beneficial bacteria.

## Introduction

Plant diseases, caused by various pathogens, including fungi, bacteria, viruses, and nematodes, affect agricultural production and lead to great yield losses. Approximately 20%–30% of yield losses in crop are caused by diseases (Savary et al., 2012; Pandin et al., 2017). To fight diseases in crops, synthetic pesticides have been widely used in the past decades. However, the excessive use of synthetic pesticides for the crops protection results in serious environmental issues and decreases agricultural sustainability (Horrigan et al., 2002; Aktar et al., 2009; Urruty et al., 2016). Nowadays, the need to explore alternative management approaches that are not only effective but also less harmful to the environment is imperative. Biological control by using beneficial organisms is a crucial alternative to mitigate the damage caused by plant diseases for a sustainable agriculture (O’Brien, 2017; Kohl et al., 2019; He et al., 2021). Bacteria from *Bacillus* genera are considered important biocontrol agents and preferred in agricultural systems because they have been proved not only to be very effective against phytopathogens, but also to be easily cultured, stored, and manufactured for biotechnological purposes due to their ability to form endospores (Nicholson, 2002; Jacobsen et al., 2004; Perez-Garcia et al., 2011; Fira et al., 2018). Increasing amount of *Bacillus*-based products has been put into application as biological pesticides and commercialized to control various pathogens of different crops/fruits/vegetables worldwide, including Serenade (*B. velezensis* QST713; AgraQuest Inc., United States), Kodiak (*B. subtilis*, Gustafson Inc., United States), Yield Shield (*B. pumilus* GB34; Bayer CropScience, United States), RhizoVital (*B. velezensis* FZB42; ABiTEP GmbH, Germany), Botrybel (*B. velezensis*; Agricaldes, Spain), Amylo-X® WG (*B. amyloliquefaciens* subsp. *plantarum* D747; Certis Europe BV, Netherlands), Symbion-P (*B. megaterium*; T. Stanes & Co. Ltd., India) (Pandin et al., 2018; Rabbee et al., 2019; Miljaković et al., 2020).

*Bacillus* spp. controls plant diseases *via* multiple mechanisms, including competition for nutrients and space with the pathogens, production of various biologically active secondary metabolites with broad spectrum antimicrobial activity such as surfactin, fengycin, or iturin, and triggering induced systemic resistance of the plants (Kloepper et al., 2004; Ongena and Jacques, 2008; Fira et al., 2018). In addition, a biocontrol strain should also fulfill the requirement for strong colonization (Niu et al., 2020; Blake et al., 2021). Bacterial cells can attach to surfaces and, after cell division and proliferation, form dense aggregates commonly referred to as biofilms. The formation of biofilms can promote microbial colonization on plant. Increasing evidence shows that the biofilms on crop surfaces may drive important bioprotection mechanisms (Chen et al., 2013; Pandin et al., 2017; Arnaouteli et al., 2021).

Biofilms are spatially structured communities of microorganisms, generally embedded in a self-produced extracellular matrix, and adhering to a living or inert surface (Vlamakis et al., 2013; Arnaouteli et al., 2021). The major components of extracellular matrix in *B. subtilis* that ensure its cohesion include protein fibers of TasA encoded by *tapA* operon, a hydrophobin-like protein BslA encoded by *bslA* and particularly exopolysaccharides (EPS). The EPS produced by *B. subtilis* is mainly attributed to *epsA-O* operon and *sacB-yveB-yveA* transcriptional unit (Branda et al., 2006; Vlamakis et al., 2013; Arnaouteli et al., 2021). Recently, an additional unknown EPS encoded by *ydaJKLMN* operon was reported to contribute to extracellular matrix and thus biofilm formation in *B. subtilis* under laboratory conditions (Kampf and Stulke, 2017; Bedrunka and Graumann, 2017b). The analysis of the contributions of the different genes revealed that the YdaL, YdaM, and YdaN proteins provide the catalytic activities for EPS synthesis and YdaJ modifies the polysaccharide. YdaK, which is also encoded within the putative EPS-synthesis operon *ydaJ-N*, is a cyclic di-GMP (c-di-GMP) binding signal transduction protein and is indispensable for EPS biosynthesis and complex colony biofilm formation in *B. subtilis* (Kampf and Stulke, 2017; Bedrunka and Graumann, 2017b). However, the function of the unknown EPS produced by YdaLMN is unclear.

C-di-GMP is a nearly ubiquitous bacterial nucleotide messenger that regulates a variety of important processes, including biofilm, antibiotic production and host colonization (Xu et al., 2018; Chou et al., 2020; Fernandez-Llamosas et al., 2021; Isenberg et al., 2022; Kharadi et al., 2022). In response to external stimuli, c-di-GMP is synthesized from 2 GTP molecules by diguanylate cyclases (DGCs) characterized by GGDEF domain. The second messenger can then bind intracellular receptors that interact with a specific target to direct physiological changes, which translates the c-di-GMP level into an adaptive cellular response. Phosphodiesterases (PDEs), characterized by EAL or HD-GYP domains, hydrolyze c-di-GMP into the linear pGpG which can be further degraded into GMP by the oligoribonucleases (Römling et al., 2013; Orr et al., 2018). Most bacterial species possess multiple DGCs and PDEs which are often involved in very specific, nonoverlapping signal transduction pathways *via* diverse c-di-GMP binding receptors (Sarenko et al., 2017; Hengge, 2021). The known classes of c-di-GMP receptors include PilZ domain proteins, proteins with degenerate GGDEF and EAL domains that can bind c-di-GMP but are enzymatically inactive, c-di-GMP-specific riboswitches, trigger PDEs, and transcriptional regulators or other proteins that do not possess a common domain organization or c-di-GMP binding motif (Chou and Galperin, 2016; Hengge, 2016). The multiplicity of DGCs and PDEs in single species and the diversity of c-di-GMP-sensing receptor components indicate that the function of c-di-GMP signaling is of unprecedented complexity.

The Gram-positive model organism *B. subtilis* possesses a relatively concise c-di-GMP signaling set. There are three DGCs, DgcK, DgcP, and DgcW, one active PDE, PdeH, and three putative c-di-GMP receptors MotI (a PilZ domain protein, also known as YpfA), YdaK (a degenerated GGDEF domain protein), and YkuI (a degenerated EAL domain protein) (Chen et al., 2012; Gao et al., 2013; Bedrunka and Graumann, 2017b; Subramanian et al., 2017; Bange and Bedrunka, 2020). At least two known c-di-GMP signaling pathways have been identified in *B. subtilis via* the receptors MotI and YdaK, respectively. One pathway represses motility *via* MotI by interacting with the flagellar stator element MotA, to disengage and sequester it from the flagellar rotor FliG (Subramanian et al., 2017). The other pathway activates the production of an unknown EPS which is synthesized by YdaLMN *via* the c-di-GMP-binding protein YdaK (Bedrunka and Graumann, 2017a,b). YkuI also serves as c-di-GMP receptor in *B. subtilis*. The concrete functional role of YkuI is still unclear, even though it has been structurally characterized in its apo- and c-di-GMP-bound states more than a decade ago in *B. subtilis* (Minasov et al., 2009).

*B. velezensis* is an important member in *B. subtilis* clade (Patel and Gupta, 2020). A number of strains within *B. velezensis* have received a lot of attention in recent years, due to their genomic robustness, and the growing evidence for their possible utilization in the agricultural industry for managing plant diseases including *B. velezensis* QST713, *B. velezensis* FZB42, *B. velezensis* SQR9 (Weng et al., 2013; Pandin et al., 2018; Adeniji et al., 2019; Rabbee et al., 2019). Evidence showed that there are two DGC, DgcK (YhcK), DgcP (YtrP), and one PDE, PdeH (YuxH) in *B. velezensis* (Yang et al., 2018). Various *B. velezensis* strains shared a high similarity of nucleotide sequence of the genes encoding c-di-GMP-metabolizing enzymes (Yang et al., 2018), suggesting that the c-di-GMP signaling pathway is conserved in *B. velezensis*. Like other bacteria, c-di-GMP also regulates swarming motility and biofilm formation in *B. velezensis* (Yang et al., 2018).

Despite extensive research, little direct evidence has been presented to demonstrate regulation of biocontrol efficacy by c-di-GMP in beneficial bacteria such as *B. subtilis* and *B. velezensis*. *B. velezensis* PG12 (formerly *B. amyloliquefaciens* PG12) is a patented strain in China isolated from apple fruit and effective to control apple ring rot disease caused by *Botryosphaeria dothidea* in the postharvest period (Chen et al., 2016a; Zeng et al., 2021). In this study, the role of c-di-GMP signaling system in biocontrol of *B. velezensis* PG12 against apple ring rot disease and its signaling pathway were investigated. We found that artificially modified c-di-GMP level in the cells of *B. velezensis* PG12 affected biofilm formation, colonization on apple fruits and its biocontrol activity against apple ring rot disease, but not the antagonistic activity of *B. velezensis* PG12 to *B. dothidea*. Evidences showed the degenerated GGDEF domain protein YdaK also regulates biofilm formation, bacterial colonization on apple fruits and biocontrol efficacy but not antagonistic activity of *B. velezensis* PG12 in the low c-di-GMP level background. Significantly, deletion of *ydaK* could rescue the inhibition of biofilm formation, colonization on apple fruits and biocontrol efficacy by low c-di-GMP level, indicating that YdaK is necessary for c-di-GMP regulation on biofilm formation, colonization on apple fruits and effective biological control. These findings suggest that c-di-GMP regulates biocontrol efficacy of *B. velezensis via* its potential receptor YdaK through regulating biofilm formation and subsequently colonization on apple fruits.

## Materials and methods

### Bacterial/fungal strains, plasmids, primers, and media

The bacterial strains and plasmids used in this study are listed in Table 1. *Bacillus velezensis* PG12 and its derivatives were stored at −80°C in 20% glycerol. *B. velezensis* strains were cultivated in Lysogeny broth (LB; 1% tryptone, 0.5% yeast extract, and 1% NaCl) or Msgg medium for biofilm formation at 30°C. The recipe for MSgg is as follows: 5 mM potassium phosphate (pH 7.0), 100 mM MOPS (morpholinepropanesulfonic acid; pH 7.0), 700 μM CaCl_2_, 2 mM MgCl_2_, 50 μM MnCl_2_, 1 μM ZnCl_2_, 50 μM FeCl_3_, 2 μM thiamine, 0.5% glutamic acid, 50 μg/ml threonine, 50 μg/ml tryptophan, 50 μg/ml phenylalanine, and 0.5% glycerol (Branda et al., 2001). *Escherichia coli* strains were cultivated in LB broth at 37°C. Antibiotics were added at the following concentrations when required: 100 μg/ml ampicillin, 20 μg/ml zeocine, 5 μg/ml erythromycin, 10 μg/ml tetracycline. Primers used for PCR in this report are summarized in Supplementary Table S1.

**Table 1:** Strains and plasmids used in this study.

Strain and plasmid	Characters^a^	Reference or source
Strains		
***Escherichia coli***		
DH5*α*	*endA1 hsdR17 supE44 thi-1 λ^−^ recA1 gyrA96 relA1* Δ*lacU169*(Φ*80dlacZ*Δ*M15*)	TakaRa
EC135	*mcrA* Δ(*mrr*-*hsdRMS*-*mcrBC*) Δ*dcm*::*FRT* Δ*dam*::*FRT*	Zhang et al. (2012)
***Bacillus velezensis***		
PG12	Wild-type strain isolated from apple	Chen et al. (2016a)
Δ*dgcK*	Δ*dgcK*::Erm^R^,	Yang et al. (2018)
Δ*dgcP*	Δ*dgcP*::Erm^R^	Yang et al. (2018)
Δ*pdeH*	Δ*pdeH*::Tet^R^	Yang et al. (2018)
Δ*ydaK*	Δ*ydaK*::Erm^R^	Yang et al. (2018)
Δ*ykuI*	*ykuI*^ΔEAL^::Tet^R^	Yang et al. (2018)
Δ*motI*	Δ*motI*::Erm^R^	This study
Δ*dgcPydaK*	Δ*dgcP*::Erm^R^*ydaK*::Tet^R^	This study
PG12(pUBXC)	pUBXC in PG12, Zeo^R^	Yang et al. (2018)
PG12(pUBX)	pUBX in PG12, Zeo^R^	Yang et al. (2018)
Δ*dgcP* (pUBX)	pUBX in Δ*dgcP*, Erm^R^,Zeo^R^	Yang et al. (2018)
Δ*ydaK*(pUBX)	pUBX in Δ*ydaK*, Erm^R^,Zeo^R^	This study
Δ*ydaK*(pUBX-*ydaK*)	pUBX-*ydaK* in Δ*ydaK*, Erm^R^,Zeo^R^	This study
PG12(pUBX-*pdeH*)	pUBX-*pdeH* in PG12, Zeo^R^	This study
Δ*dgcP*(pUBX-*pdeH*)	pUBX-*pdeH* in Δ*dgcP*, Erm^R^,Zeo^R^	This study
Δ*pdeH*(pUBX-*dgcP*)	pUBX-*dgcP* in Δ*pdeH*, Tet^R^,Zeo^R^	Yang et al. (2018)
Δ*ydaK*(pUBX-*pdeH*)	pUBX-*dgcP* in Δ*ydaK*, Erm^R^,Zeo^R^	This study
Δ*ykuI* (pUBX-*pdeH*)	pUBX-*dgcP* in Δ*ykuI*, Tet^R^,Zeo^R^	This study
Δ*motI*(pUBX-*pdeH*)	pUBX-*dgcP* in Δ*motI*, Erm^R^,Zeo^R^	This study
Δ*dgcPydaK*(pUBX-*pdeH*)	pUBX-*dgcP* in Δ*dgcPydaK*, Tet^R^,Erm^R^,Zeo^R^	This study
**Plasmids**		
pWYE782	*gp35* cloned into pAX01, Em^R^	Sun et al. (2015)
pMD19-T	TA cloning vector, Amp^R^	TakaRa, Dalian, China
pGFP78	pHY300PLK containing GFP gene, Amp^R^ and Tet^R^	Zhang et al. (2017)
pMDT3	pMD19-T containing the Tet^R^ gene from pGFP78, Amp^R^ and Tet^R^	Fan et al. (2017)
pMDE-*motI*	pMDE containing *motI* upper and lower arm fragment, Erm^R^, Amp^R^	This study
pMDT3-*ydaK*	pMDT3 containing *ydaK* upper and lower arm fragment, Tet^R^, Amp^R^	Yang et al. (2018)
pUBX	*Bacillus*-*Escherichia coli* shuttle vector containing *PxylA*, *xylR*, t0 transcription terminator, Zeo^R^	Chen et al. (2016b)
pUBXC	pUBX containing *comK*_Bsu_ under the control of xylose inducible system, Zeo^R^	Chen et al. (2016b)
pUBX-*pdeH*	pUBX containing *pdeH* under the control of xylose inducible system, Zeo^R^	Yang et al. (2018)
pUBX-*dgcP*	pUBX containing *dgcP* under the control of xylose inducible system, Zeo^R^	Yang et al. (2018)
pUBX-*ydaK*	pUBX containing *ydaK*_PG12_ under the control of xylose inducible system, Zeo^R^	This study

^a^Erm^R^, Tet^R^, Zeo^R^, Amp^R^ stand for resistance to erythromycin, tetracycline, zeocine and ampicillin, respectively.

*Botryosphaeria dothidea* YL1, a virulent strain caused apple ring rot disease, was supplied by Prof. Liyun Guo from China Agricultural University. *B. dothidea* YL1 was cultured on potato dextrose agar (PDA) plate at 25°C for 1 week in the dark.

### Strain construction

The *motI* deletion mutant was constructed by marker exchange mutagenesis. The recombinant plasmid pMDE-*motI* was firstly generated and *E. coli* strain DH5*α* was used as a cloning host for plasmid construction. Upstream and downstream fragments flanking the *motI* gene were amplified by polymerase chain reaction (PCR) using genomic DNA of *B. velezensis* PG12 as template and primers Up-F/Up-R and Dn-F/Dn-R, respectively. An erythromycin cassette, amplified from pAX01-gp35 (Table 1), was ligated with these two fragments and then cloned into pMD19-T to generate pMDE-*motI*. This construct was transformed into *E. coli* strain EC135 lacking all of the known Restriction-Modification (R-M) systems for demethylation modification to increase the genetic transformation efficiency into *B. velezensis* PG12, because R-M systems are believed to act as defenses to protect the prokaryotic cells against invading DNA and exogenous DNA with foreign methylation patterns are recognized and rapidly degraded, which hinders the experimental genetic manipulation of many bacteria species such as *B. velezensis* (Zhang et al., 2012).

The demethylated pMDE-*motI* was introduced into *B. velezensis* PG12 harboring pUBXC through natural genetic competence to generate Δ*motI* as described previously with slight modifications (Chen et al., 2016b). In brief, the *Bacillus*-*E. coli* shuttle vector pUBXC carrying the xylose-inducible *comK* expression cassette was transformed into *B. velezensis* PG12 by electroporation firstly. The cells carrying pUBXC cultured at 37°C with shaking at 200 rpm until the OD_600_ reached to 0.5, followed by adding a final concentration of 0.2% xylose to the culture to artificially induce the genetic competence (Chen et al., 2016b). After 1 h incubation at 37°C with shaking at 170 rpm, 200 μl competent cells were taken to mix with 10 μl demethylated pMDE-*motI* in a new 2-ml tube and incubated at 37°C with shaking at 120 rpm for another 3 h to introduce demethylated pMDE-*motI* into *B. velezensis* PG12 through natural competence. Finally, cells were plated on LB agar plates containing erythromycin. After PCR and DNA sequencing verification with primers *motI*-in-F/R and *motI*-out-F/R, pUBXC was kicked off by successive culture in LB without zeocine and selected based on sensitivity to 20 mg/ml zeocine. *dgcP* and *ydaK* double deletion mutant (Δ*dgcPydaK*) was constructed in the same way by using the recombinant plasmid pMDT3-*ydaK* in the background of Δ*dgcP*.

To construct low c-di-GMP level strains, pUBX-*pdeH* was extracted from *E. coli* stain EC135 and electroporated into *B. velezensis* PG12 or its derived mutants. To construct the complementary strain Δ*ydaK*(pUBX-*ydaK*), the plasmid pUBX-*ydaK* was firstly constructed. The open reading frame of *ydaK* was amplified by PCR and cloned into pUBX to generate pUBX-*ydaK*. After PCR and sequencing verification with primers pUBX-F/pUBX-R, the construct was electroporated into *E. coli* strain EC135 strain and subsequently into Δ*ydaK*. Transformants were then selected on 20 mg/ml zeocine-containing LB plates and verified by PCR and DNA sequencing.

### Biocontrol assay against apple ring rot disease

Fuji apple fruits were used to evaluate the biocontrol activity of *B. velezensis* PG12 and its derivatives against apple ring rot disease. The bacteria were cultivated in LB broth in a shaker overnight at 30°C in the test tubes and then transferred to 200 ml LB broth in 500-mL flask and incubate for 48 h. 1% xylose solution at the final concentration was added when the overexpression strain was cultured. When required, cell-free supernatants were obtained after centrifugation at 6,000 × *g* for 20 min at 4°C, and then filtration using a 0.22-μm pore size filter. The cultures or supernatants were diluted twice when used. Fuji apples with the same size and maturity and no wound were selected, 20 apples for each treatment. After surface disinfection, the apples were placed in the hood for drying. Then, the apples were soaked in the prepared cultures or supernatants for 1 h and dried in the hood, and then placed in the plastic trays. Apple fruits soaked with the LB broth were used as controls. After 24 h incubation at 25°C in the chamber, 5-mm diameter mycelia disks of the pathogen were placed on the wounds which were made by a bunch of pins on apple fruits. Each apple fruit was inoculated with four fungal disks. The inoculated apple fruits were put back to the plastic trays with 90% humidity and incubated in the chamber under a 12 h photoperiod. Images were taken using a Nikon BM-7 digital camera (Nikon Corporation, Tokyo, Japan) and the diameter of disease lesions was measured 6–8 days post inoculation. Four fruits were used for each replicate and five replicates were set for each treatment. Three independent biological repeats were conducted.

### *In vitro* antagonism test

The antifungal activity of bacteria culture or cell-free supernatant against *B. dothidea* was explored using the dual culture assay. Briefly, a 5-mm diameter mycelial disc (5-day-old) of *B. dothidea* was placed in the center of fresh PDA plates (90 mm). 1% xylose solution at the final concentration was added to the PDA medium when poured into the plates. Four drops of 1-μl bacterial cultures or 200 μl cell-free supernatants were equidistantly spotted around the fungal inoculums at a distance of 2.25 cm. LB broth was used as control. Plates were incubated at 25°C for 3 days, and the antagonistic effect was assessed by measuring the inhibition zone. The experiments were repeated twice, and results were recorded as the mean of three replicates.

### Biofilm formation assay

The biofilm formation experiment was performed by using the microtiter plates as described previously with minor modification (Gao et al., 2015). *B. velezensis* cells were grown in LB broth at 37°C overnight and then transferred to fresh LB broth to grow to mid-log phase (OD_600_ 0.8–1.0). 2 μl of the cultures mixed with 2 ml of MSgg broth were transferred to the wells of 24-well microtiter plates. 1% xylose solution at the final concentration was added to MSgg broth. The plates were incubated at 28°C for 48–72 h. Images were taken using a Nikon BM-7 digital camera (Nikon Corporation, Tokyo, Japan). Four replicates were set for each treatment. Three independent biological repeats were conducted.

### Assay of bacterial colonization on apple fruits

To study the effect of c-di-GMP on *B. velezensis* colonization on apple fruits, cell population of *B. velezensis* PG12(pUBX) and its derivatives on the surface of apple fruits was monitored on the 2nd and 7th day after inoculation. Briefly, the bacteria were cultivated in LB broth in a shaker overnight at 30°C in the test tubes and then transferred to 200 ml LB broth in 500-ml flask and incubate for 48 h. 1% xylose solution at the final concentration was added when cultured. The cell concentration was adjusted to 2.5 × 10^8^ cells/ml to use. Fuji apples with the same size and maturity and no wound were selected. After surface disinfection, the apples were placed in the hood for drying. Then, the apples were soaked in the prepared cultures for 1 h and dried in the hood, and then placed in the plastic trays. Apple fruits soaked with the LB broth were used as controls. Three apples were used for each treatment. 7.5 cm^2^ pieces were taken from the surface of each apple fruit at the 2nd and 7th day after inoculation, respectively, and then disrupted in a sterile mortar and pestle. The suspensions were diluted by 10-fold serial dilutions in sterile water, and 100 ml of each diluted suspension was plated on LB agar plates supplemented with zeocine and then incubated at 30°C. Bacterial colony-forming units (CFU) on each plate were counted and the collected data were analyzed. The experiments were repeated twice.

### Extraction and quantification of the intracellular c-di-GMP

Extraction of c-di-GMP was performed as described previously (Yang et al., 2018). In brief, *B. velezensis* strains were cultured in LB broth at 37°C and 1% (w/v) xylose at the final concentration was added. The cells were grown to an OD_600_ of 0.8 and 50 ml cultures were precipitated by centrifugation at 4°C. The cell pellet was resuspended in 1 ml ice-cold extraction solvent (a mixture of acetonitrile-methanol–water; 40:40:20 by volume) to quench metabolism and initiate the extraction process followed by a 15-min incubation step at 4°C. The cell suspension was then heated to 95°C for 10 min. After cooling, the suspension was centrifuged at 14,000 rpm for 5 min. The upper soluble phase was transferred to a new Eppendorf tube, while the resulting pellets was extracted twice as above with 500 μl extraction solvent at 4°C. The extracted samples were stored at −80°C before LC–MS/MS analysis.

The intracellular c-di-GMP level was determined by using an Agilent 1290 (Agilent, United States) liquid chromatograph (LC) equipped with a QQQ (Agilent 6460, United States) tandem mass spectrometer (LC–MS/MS) in Tsinghua University (Yang et al., 2018). A XSelect HSS T3 2.5 μm, 2.1 × 100 mm (waters United States) was used for LC separation, using a gradient elution of methanol as solvent A and 10 mM tributylamine and 15 mM glacial acetic acid diluted in water as solvent B. The gradient program was as follows: 0–2 min 5% A, 2–4 min 5% A to 60% A, 4–5.5 min 60% A to 100% A,5.5–6.5 min 100% A. 6.5–6.6 min 100% A to 5% A, 6.6–10 min 5% A. The flow rate was 0.3 ml min^−1^ and the injection volume was 10 μl. The total run time was 10 min for each sample. The negative ion mode was used to detect c-di-GMP. The mass spectrometer was operated in both electrospray ionization mode and in multiple reaction monitoring mode. The nebulizer was set at 40 psi and the capillary was set at −3,500 V. High-purity nitrogen served as both the nebulizing and dry gas. The gas temperature was held at 350°C and the gas flow was 8 l min^−1^. Chemically synthesized c-di-GMP (BIOLOG life science institute, Bremen, Germany) was dissolved in the extraction buffer and serially diluted to generate a standard curve for calculating the c-di-GMP concentration in each extract.

Three replicates were set for each treatment and the results were recorded as the mean of three replicates. The experiments were repeated twice.

### Statistical analysis

Analysis of variance (ANOVA) was performed with the statistical program SPSS software 21.0 using the least significant difference (LSD) test to assess significant differences between treatments (*p* < 0.05).

## Results

### Deletion of a single diguanylate cyclase or phosphodiesterase encoding gene does not affect biocontrol efficacy of *B. velezensis* against apple ring rot

**Figure 1:**
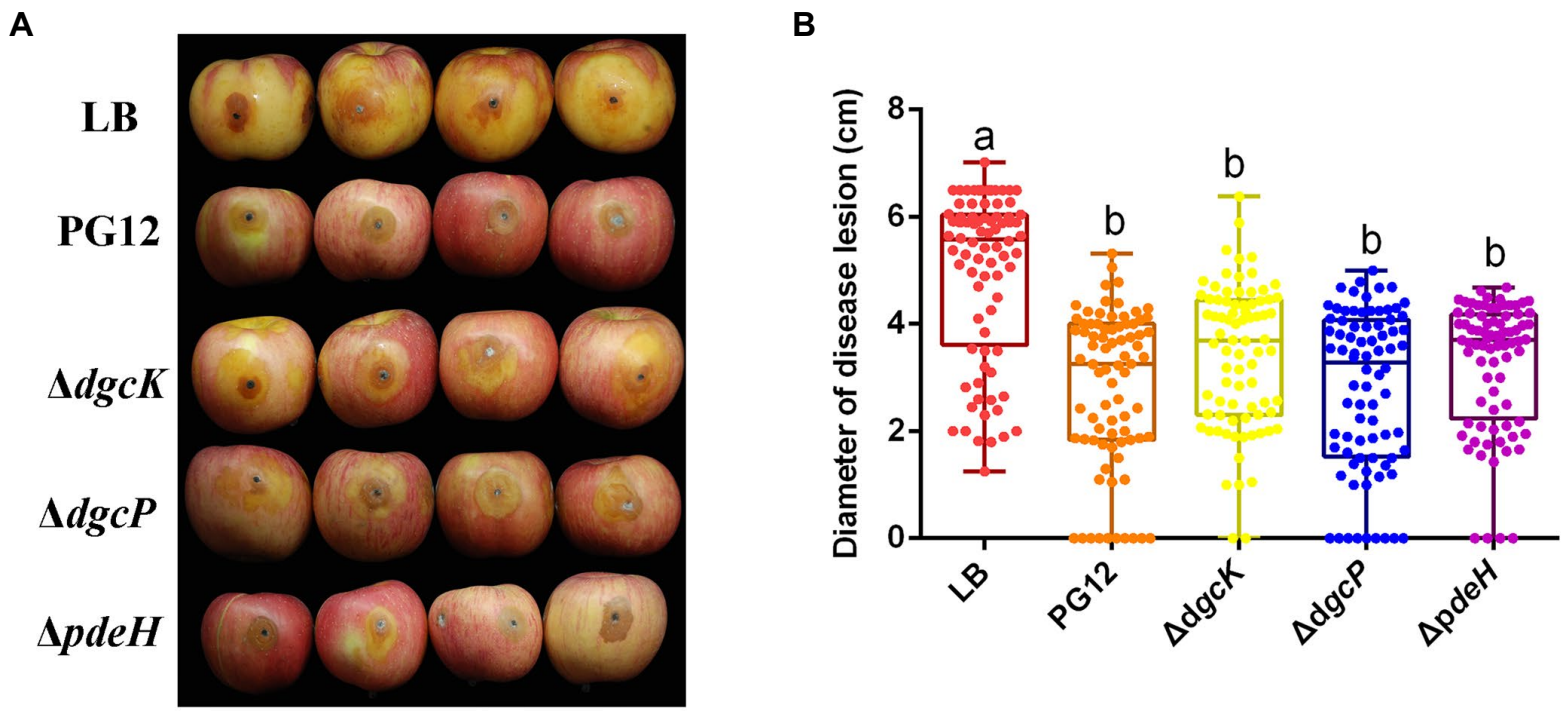
Single deletion of diguanylate cyclase or phosphodiesterase encoding gene does not affect the biocontrol efficacy of *B. velezensis* against apple ring rot disease. Bacteria were incubated for 48 h in LB broth. Biocontrol efficacy was assessed with Fuji apple fruits treated with the 48-h cultures of *B. velezensis* PG12 (PG12), Δ*dgcK*, Δ*dgcP* or Δ*pdeH*. Apple fruits soaked with the LB broth (LB) were used as controls. Images were taken **(A)** and the diameter of disease lesions **(B)** was measured 8 days post inoculation. Values correspond to the mean diameter of 80 disease lesions from 20 apple fruits. The data is a representative of three independent experiments. Different letters on the column indicate significant differences between different treatments according to the least significant difference (LSD) test (*p* < 0.05).

To test whether c-di-GMP is involved in the biocontrol efficacy of PG12 against apple ring rot disease, the biocontrol activity of the DGC or PDE single gene deletion mutant was investigated. As expected, the size of disease lesions on apples treated with the culture of PG12 was significantly reduced compared to the LB-treated control, indicating the significant biocontrol efficacy of PG12 against apple ring rot disease (Figure 1). However, the disease lesions of apples treated with Δ*dgcK*, Δ*dgcP*, or Δ*pdeH* showed no significant difference with that of the wild-type PG12 (Figure 1). These results indicate that single deletion of a DGC or PDE encoding gene does not affect the biocontrol efficacy of PG12 against apple ring rot disease.

### Overexpression of a diguanylate cyclase or a phosphodiesterase affects biocontrol efficacy of *B. velezensis* against apple ring rot

**Figure 2:**
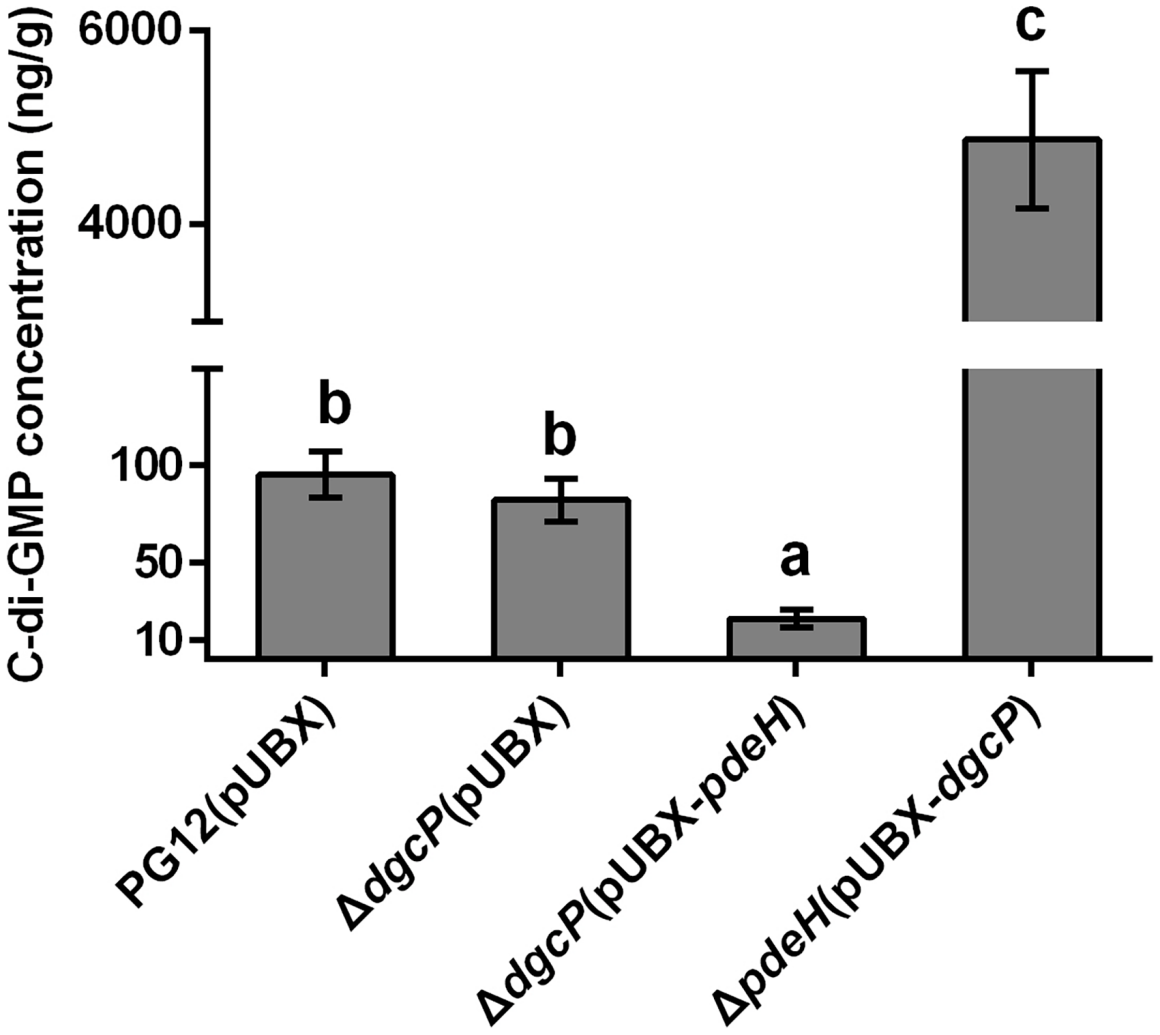
Measurement of the intracellular c-di-GMP level in *B. velezensis* PG12 (pUBX) and its derived strains. *B. velezensis* PG12(pUBX) [PG12(pUBX)], Δ*dgcP*(pUBX), Δ*dgcP*(pUBX-*pdeH*), and Δ*pdeH*(pUBX-*dgcP*) were cultured in LB broth with 1% (w/v) xylose at the final concentration at 37°C. The extraction was conducted when the cells were grown to an OD_600_ of 0.8. The intracellular c-di-GMP level was determined using LC–MS/MS. Values correspond to the mean concentration of three replicates. The data is a representative of two independent experiments. Different letters on the column indicate significant differences between different treatments according to the least significant difference (LSD) test (*p* < 0.05).

**Figure 3:**
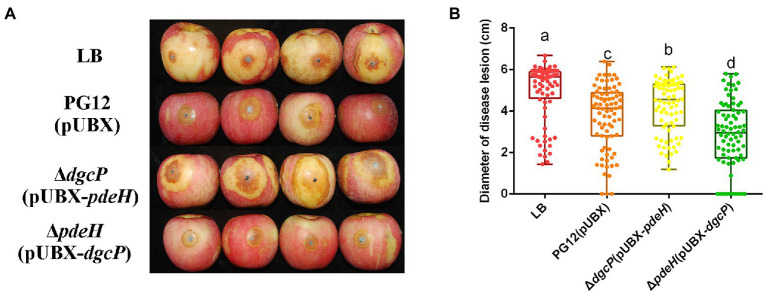
Artificially elevated or reduced c-di-GMP changes the biocontrol efficacy of *B. velezensis* against apple ring rot disease. Bacteria were incubated for 48 h in LB broth supplemented with 1% (w/v) xylose at the final concentration. Biocontrol efficacy was assessed with Fuji apple fruits treated with the cultures of *B. velezensis* PG12(pUBX) [PG12(pUBX)], Δ*dgcP*(pUBX-*pdeH*) or Δ*pdeH*(pUBX-*dgcP*). Apple fruits soaked with the LB broth (LB) were used as controls. Images were taken **(A)** and the diameter of disease lesions **(B)** was measured 7 days post inoculation. Values correspond to the mean diameter of 80 disease lesions from 20 apple fruits. The data is a representative of three independent experiments. Different letters on the column indicate significant differences between different treatments according to the least significant difference (LSD) test (*p* < 0.05).

As our previous results showed that there was no significant difference in the c-di-GMP concentration between PG12 and Δ*dgcK*/Δ*dgcP* (Yang et al., 2018), we expect that single deletion of a DGC gene leads to no change of c-di-GMP level to affect the biocontrol efficacy of PG12 against apple ring rot disease. Even though the c-di-GMP concentration in Δ*pdeH* was ~35-fold higher than that in PG12 (Yang et al., 2018), deletion of *pdeH* did not cause significant change in the biocontrol activity compared with the wild-type PG12 (Figure 1). We speculate that there are both low and high thresholds of c-di-GMP levels to change the biocontrol activity. The c-di-GMP level in Δ*pdeH* is probably below the high threshold and could not affect its biocontrol efficacy. To further investigate whether higher or lower concentration of c-di-GMP affects biocontrol efficacy of PG12 or not, strains Δ*dgcP*(pUBX-*pdeH*) and Δ*pdeH*(pUBX-*dgcP*) were constructed and the biocontrol efficacy was detected. Our results showed the c-di-GMP concentration in Δ*dgcP*(pUBX-*pdeH*) was 4.6-fold lower than that in PG12, whereas it was 51-fold higher in Δ*pdeH*(pUBX-*dgcP*) than that in PG12 (Figure 2). The biocontrol assay against apple ring rot disease showed that the size of disease lesions of apples treated with Δ*dgcP*(pUBX-*pdeH*) is significantly higher than that with PG12(pUBX), while the size of disease lesions of apples treated with Δ*pdeH*(pUBX-*dgcP*) was significantly lower than that with PG12 (pUBX) (Figure 3). These results indicate that c-di-GMP positively regulates biocontrol efficacy against apple ring rot in PG12 and the biocontrol efficacy is more sensitive to low c-di-GMP level rather than high c-di-GMP level.

### C-di-GMP does not affect the antagonistic activity of *B. velezensis* against *Botryosphaeria dothidea*

**Figure 4:**
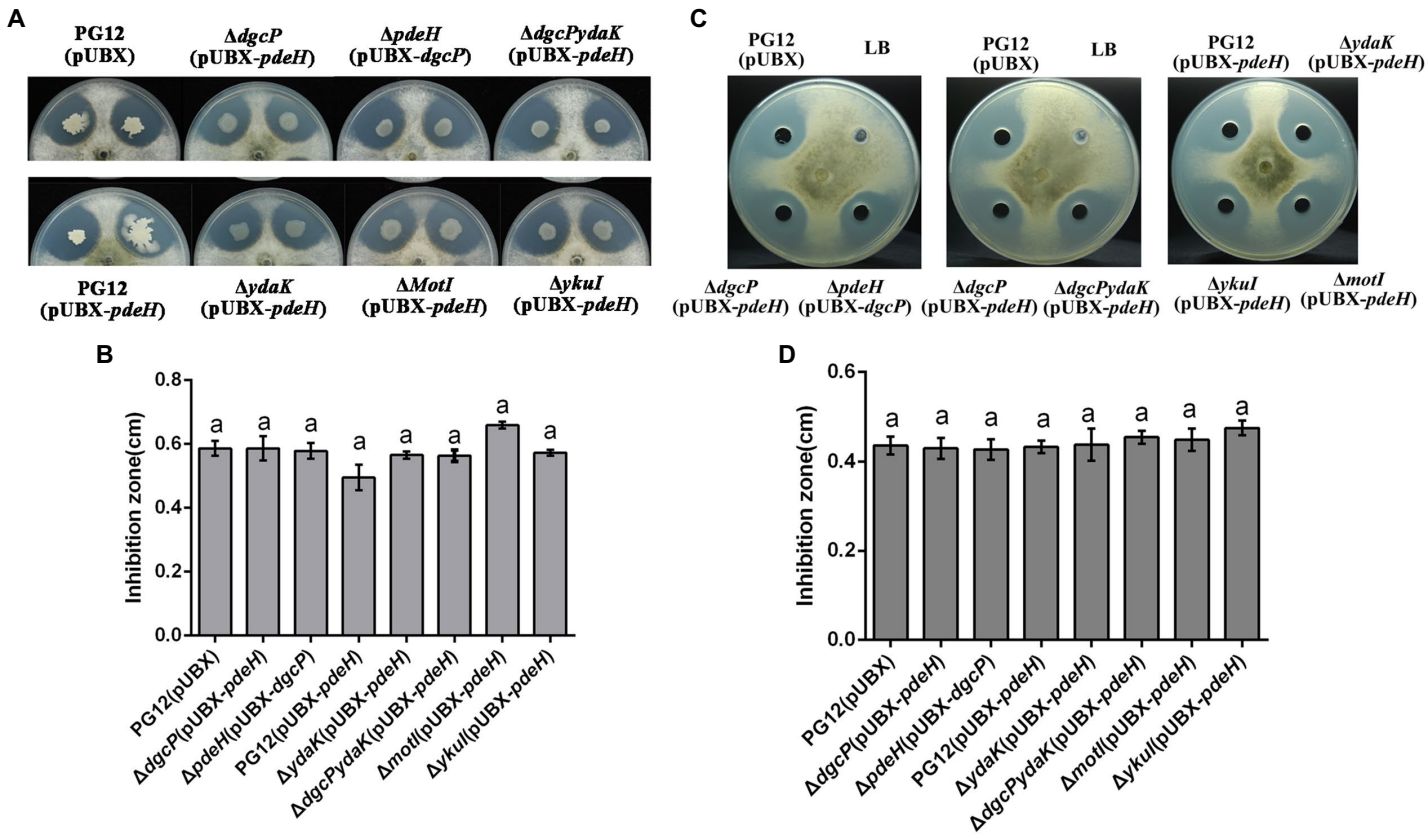
The antagonistic activity of *B. velezensis* PG12(pUBX) and its derivatives against apple ring rot disease. The antifungal activity of bacterial cultures **(A,B)** or cell-free supernatants **(C,D)** against *B. dothidea* was explored using the dual culture assay in PDA plates supplemented 1% xylose solution at the final concentration. 1-μl bacterial cultures or 200 μl cell-free supernatants of *B. velezensis* PG12(pUBX) [PG12(pUBX)], PG12(pUBX-*pdeH*) [PG12(pUBX-*pdeH*)], Δ*dgcP*(pUBX-*pdeH*), Δ*pdeH*(pUBX-*dgcP*), Δ*ydaK*(pUBX-*pdeH*), Δ*motI* (pUBX-*pdeH*), Δ*ykuI* (pUBX-*pdeH*), or Δ*dgcPydaK*(pUBX-*pdeH*) were equidistantly spotted around the fungal inoculums. LB broth (LB) was used as control. Images were taken **(A,C)** and the diameter of disease lesions **(B,D)** was measured 7 days post inoculation. Values correspond to the mean inhibition zone of three replicates. The data is a representative of three independent experiments. Different letters on the column indicate significant differences between different treatments according to the least significant difference (LSD) test (*p* < 0.05).

Since PG12 showed remarkable antifungal activity against *B. dothidea in vitro* (Chen et al., 2016a) and c-di-GMP regulates antibiotic production in some bacteria (Xu et al., 2018), the antagonistic activity was firstly investigated to interpret the c-di-GMP signaling pathway in regulating biocontrol activity of PG12 against apple ring rot disease. The dual culture assay showed strong inhibition of PG12(pUBX) on *B. dothidea* as expected (Figure 4). However, no significant difference of the inhibition zone between PG12(pUBX) and Δ*dgcP*(pUBX-*pdeH*)/Δ*pdeH*(pUBX-*dgcP*) was observed (Figure 4), indicating that c-di-GMP does not affect the antagonistic activity of PG12 against *B. dothidea in vitro*.

**Figure 5:**
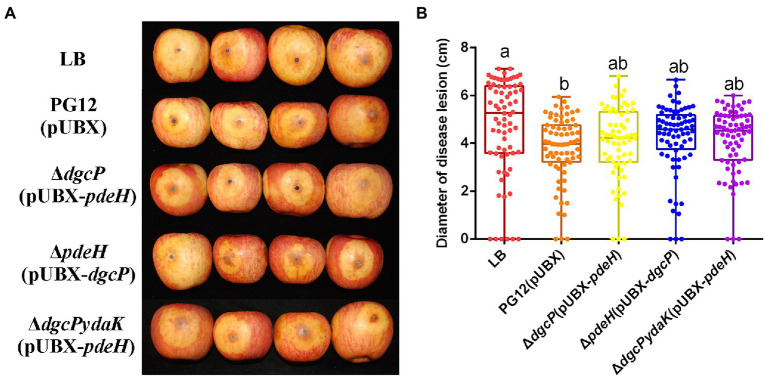
The biocontrol efficacy of the supernatants of *B. velezensis* PG12(pUBX) and its derivatives against apple ring rot disease. Bacteria were incubated for 48 h in LB broth supplemented with 1% (w/v) xylose at the final concentration. The cell-free supernatants were obtained after centrifugation at 6,000 ×g for 20 min at 4°C, and then filtration using a 0.22-μm pore size filter. Biocontrol efficacy was assessed with Fuji apple fruits treated with the supernatants of *B. velezensis* PG12(pUBX) [PG12(pUBX)], Δ*dgcP*(pUBX-*pdeH*), Δ*pdeH*(pUBX-*dgcP*), or Δ*dgcPydaK*(pUBX-*pdeH*). LB broth (LB) was used as control. Images were taken **(A)** and the diameter of disease lesions **(B)** was measured 7 days post inoculation. Values correspond to the mean diameter of 80 disease lesions from 20 apple fruits. The data is a representative of three independent experiments. Different letters on the column indicate significant differences between different treatments according to the least significant difference (LSD) test (*p* < 0.05).

To test whether c-di-GMP signaling pathway affects the antifungal activity of PG12 against *B. dothidea in vivo*, biocontrol assay was further conducted by using the supernatant of the culture of PG12(pUBX) and its derivatives on apples. The data showed that there was no significant difference in the size of disease lesions on apples pre-treated with Δ*dgcP*(pUBX-*pdeH*) or Δ*pdeH*(pUBX-*dgcP*) supernatant compared to that with PG12(pUBX) supernatant (Figure 5), indicating that c-di-GMP does not affect the antifungal activity of PG12 against the pathogen *B. dothidea in vivo*.

All these results above suggest that c-di-GMP regulation on biocontrol activity of PG12 is not through its antifungal activity against *B. dothidea*.

### YdaK regulates biocontrol efficacy of *B. velezensis* against apple ring rot disease

**Figure 6:**
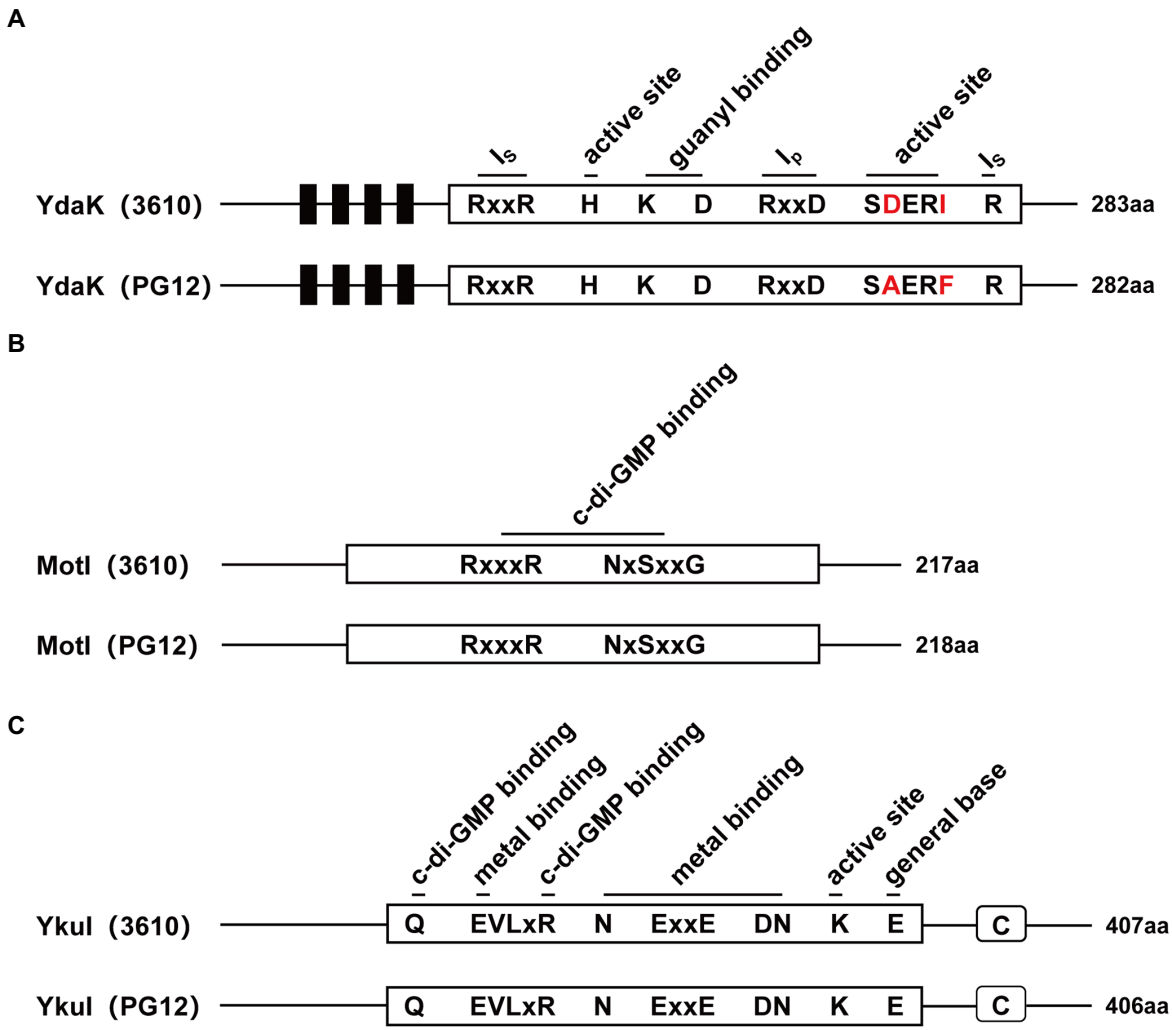
Domain architectures of Ydak, MotI, and YkuI in *B. velezensis* PG12 and *B. subtilis* 3,610. The number of amino acids in each protein is shown. **(A)** The domain architecture of the degenerated GGDEF domain of YdaK. Ip is the primary inhibitory binding site for c-di-GMP and is the secondary inhibitory binding site for c-di-GMP. Active site means the degenerated GGDEF. Predicted transmembrane regions are shown as black bars. **(B)** The domain architecture of the PilZ domain protein MotI with highly conserved c-di-GMP binding site. **(C)** The domain architecture of the degenerated EAL domain of YkuI. Highly conserved sites of the degenerated EAL residues are outlined in black. C stands for C-terminal domain from YkuI of unknown function.

**Figure 7:**
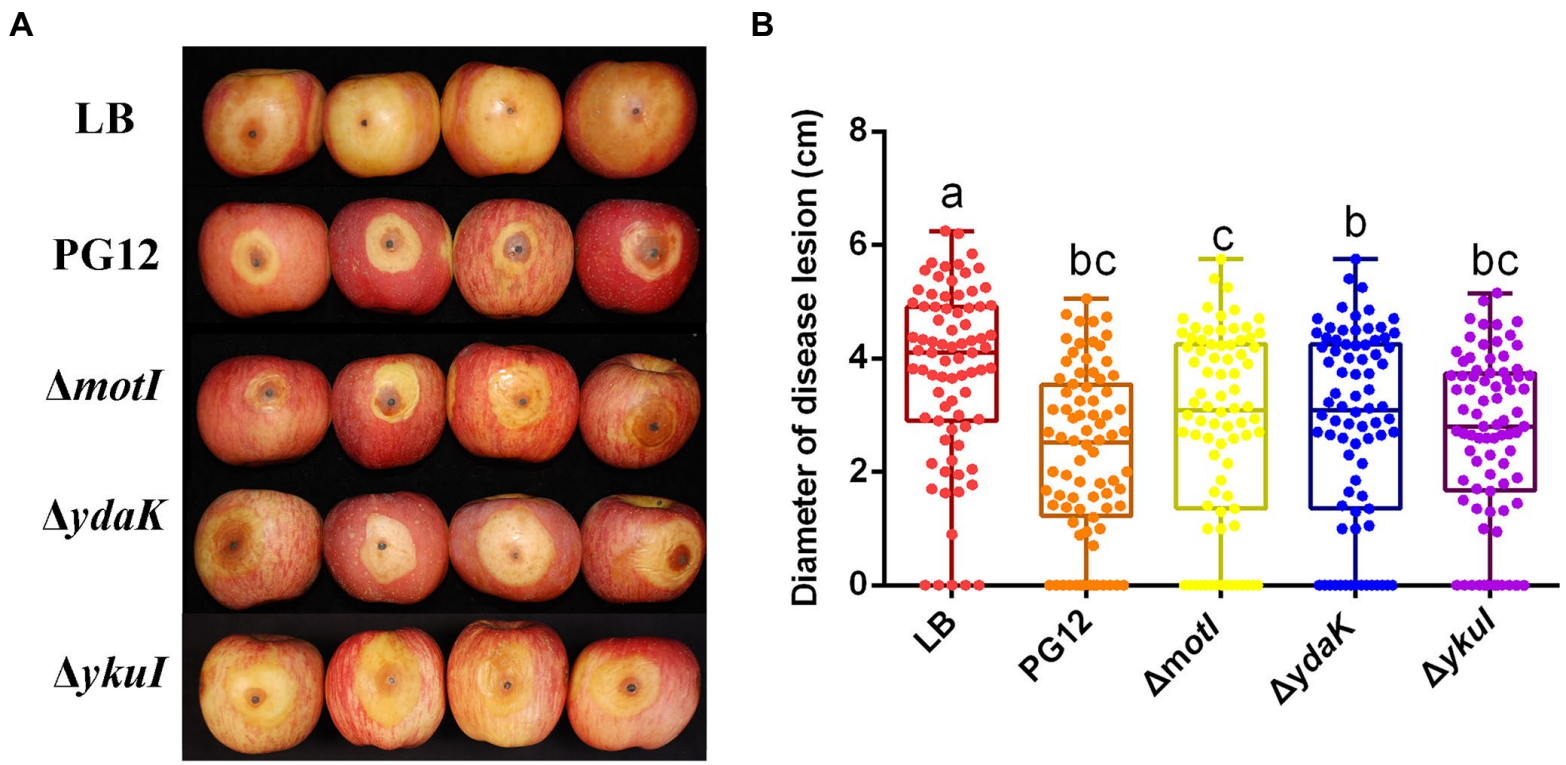
Deletion of the potential c-di-GMP receptors does not affect the biocontrol efficacy of *B. velezensis* against apple ring rot disease. Bacteria were incubated for 48 h in LB broth. Biocontrol efficacy was assessed with Fuji apple fruits treated with the cultures of *B. velezensis* PG12(PG12), Δ*motI*, Δ*ydaK*, or Δ*ykuI*. LB broth (LB) was used as control. Images were taken **(A)** and the diameter of disease lesions **(B)** was measured 7 days post inoculation. Values correspond to the mean diameter of 80 disease lesions from 20 apple fruits. The data is a representative of three independent experiments. Different letters on the column indicate significant differences between different treatments according to the least significant difference (LSD) test (*p* < 0.05).

**Figure 8:**
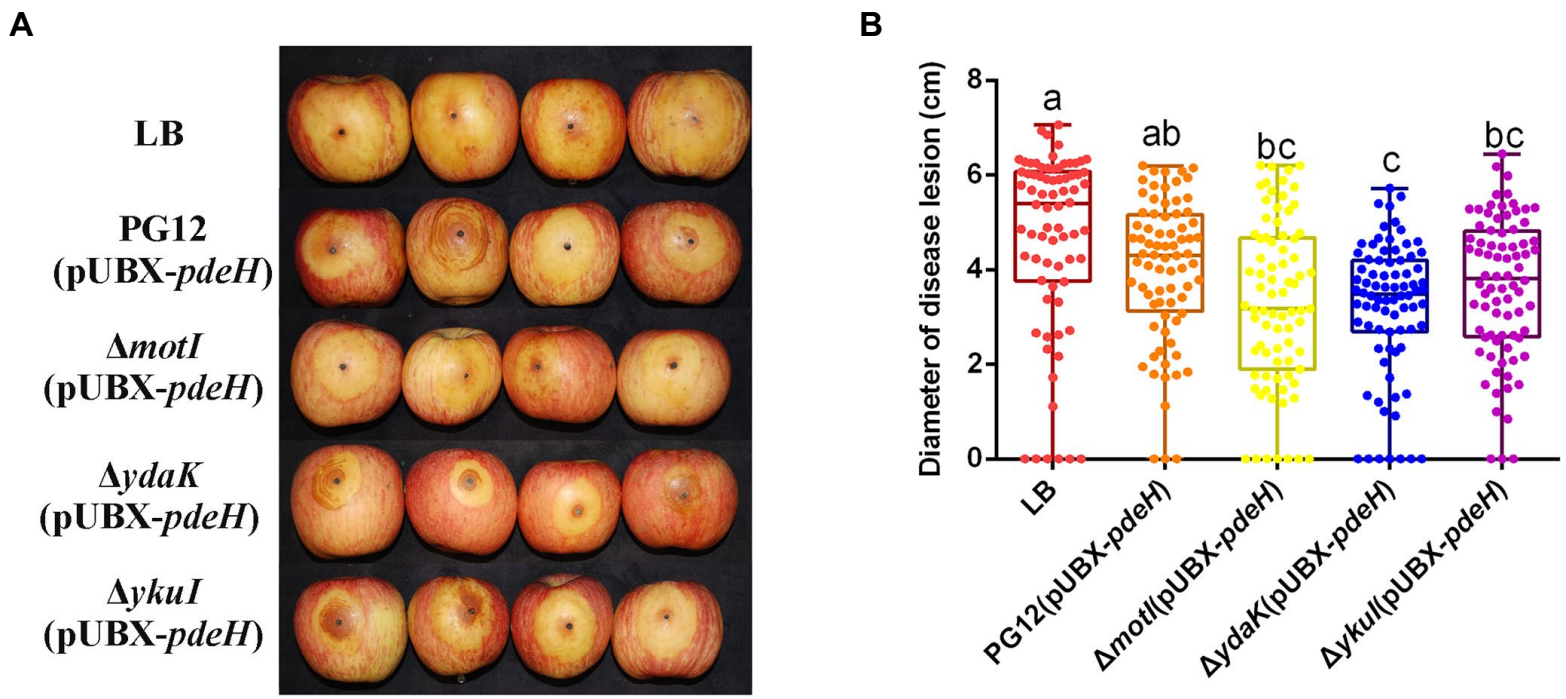
Effect of the potential c-di-GMP receptors on biocontrol efficacy against apple ring rot disease in low c-di-GMP background. Bacteria were incubated for 48 h in LB broth supplemented with 1% (w/v) xylose at the final concentration. Biocontrol efficacy was assessed with Fuji apple fruits treated with the cultures of *B. velezensis* PG12(pUBX-*pdeH*) [PG12(pUBX-*pdeH*)], Δ*motI*(pUBX-*pdeH*), Δ*ydaK*(pUBX-*pdeH*), or Δ*ykuI*(pUBX-*pdeH*). LB broth (LB) was used as control. Images were taken **(A)** and the diameter of disease lesions **(B)** was measured 7 days post inoculation. Values correspond to the mean diameter of 80 disease lesions from 20 apple fruits. The data is a representative of three independent experiments. Different letters on the column indicate significant differences between different treatments according to the least significant difference (LSD) test (*p* < 0.05).

Biofilm formation and motility are two bacterial traits which are crucial for successful colonization of a beneficial bacterium and therefore great important for its biocontrol efficacy (Compant et al., 2010; Raaijmakers et al., 2010; Cui et al., 2019; Blake et al., 2021). Since c-di-GMP regulates biofilm formation and motility in PG12 (Yang et al., 2018) but does not regulate its antifungal activity as shown above, we hypothesize that c-di-GMP regulates biofilm formation and/or motility and further biocontrol efficacy of PG12 against apple ring rot disease. MotI and YdaK serve as c-di-GMP receptors in regulating flagellar motility and an unknown EPS affecting macro colony architecture in *B. subtilis*, respectively (Subramanian et al., 2017; Bedrunka and Graumann, 2017a,b). Bioinformatic analysis showed that MotI and YdaK in PG12 shared 55% and 71% amino acid identity with those in *B. subtilis* NCIB 3610, respectively (Supplementary Figure S1). Importantly, the degenerated GGEDF domain in YdaK and the PliZ domain in MotI are conserved in *B. subtilis* NCIB 3610 and PG12 (Figures 6A,B). Therefore, we hypothesize that they share similar function in PG12 to that in *B. subtilis*. To test whether c-di-GMP regulates biofilm formation and/or motility and further biocontrol efficacy of PG12 against apple ring rot disease, deletion mutants Δ*motI* and Δ*ydaK* were generated and their biocontrol efficacy to apple ring rot disease was firstly detected. The biocontrol assay showed that there was no significant difference in the size of disease lesions on apples when they were pre-treated with PG12, Δ*motI* or Δ*ydaK* (Figure 7). Since the biocontrol efficacy of PG12 against apple ring rot is more sensitive to low c-di-GMP level, the impact of *motI* or *ydaK* on the biocontrol efficacy was further explored in the low c-di-GMP level background in which *pdeH* was overexpressed. The size of disease lesions on apples pre-treated with Δ*motI*(pUBX-*pdeH*) showed no significant difference with that of PG12(pUBX-*pdeH*) (Figure 8). Surprisingly, the size of disease lesions on apples pre-treated with Δ*ydaK*(pUBX-*pdeH*) was significantly reduced compared to that with PG12(pUBX-*pdeH*) (Figure 8). These results suggest that YdaK regulates biocontrol efficacy of PG12 against apple ring rot disease in the low c-di-GMP level background.

YkuI also serves as c-di-GMP receptor in *B. subtilis*, even though the concrete functional role of YkuI is still mysterious. The bioinformatic analysis showed that YkuI in PG12 shared 87% amino acid identity with that in *B. subtilis* NCIB 3610 (Supplementary Figure S1) and the degenerated EAL domain in YkuI is conserved in *B. subtilis* NCIB 3610 and *B. velezensis* PG12 (Figure 6C). Therefore, we speculate that YkuI also functions as c-di-GMP receptor in PG12. To test whether YkuI regulates biocontrol activity of PG12, deletion mutant Δ*ykuI* was also generated and its biocontrol efficacy to apple ring rot disease was evaluated. The biocontrol assay showed that deletion of *ykuI* did not lead to significant changes in the size of disease lesions on apples no matter in the wild-type background (Figure 7) or the low c-di-GMP level background (Figure 8), suggesting that YkuI does not affect the biocontrol efficacy of PG12 against apple ring rot and c-di-GMP regulates biocontrol efficacy of PG12 against apple ring rot disease in a YkuI-independent manner.

Altogether, these results suggest that YdaK, but not MotI or YkuI, regulates biocontrol efficacy of PG12 against apple ring rot disease.

### C-di-GMP regulates biocontrol efficacy of *B. velezensis* through the potential receptor YdaK

**Figure 9:**
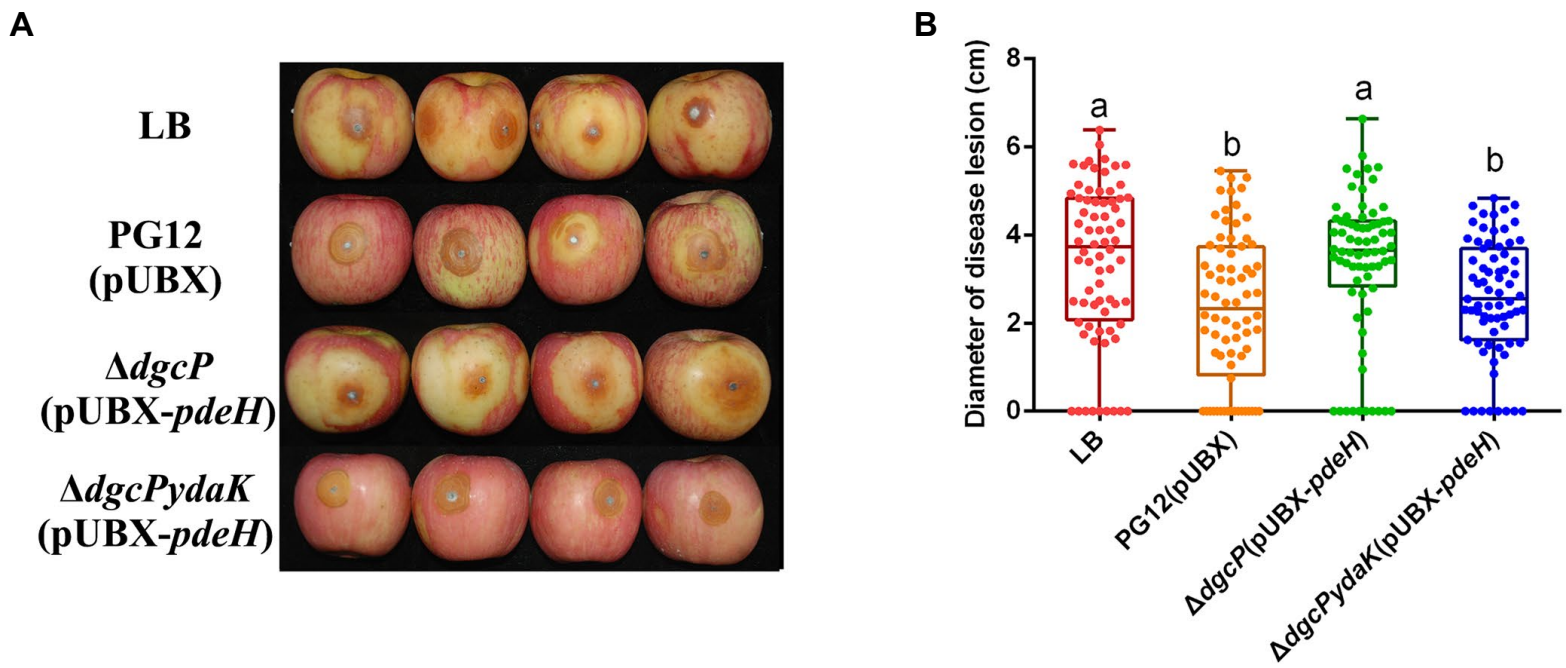
Deletion in the *ydaK* gene rescued the inhibition of biocontrol efficacy by low c-di-GMP level. Bacteria were incubated for 48 h in LB broth supplemented with 1% (w/v) xylose at the final concentration. Biocontrol efficacy was assessed with Fuji apple fruits treated with the cultures of *Bacillus velezensis* PG12(pUBX) [PG12(pUBX)], Δ*dgcP*(pUBX-*pdeH*), or Δ*dgcPydaK*(pUBX-*pdeH*). LB broth (LB) was used as control. Images were taken **(A)** and the diameter of disease lesions **(B)** was measured 7 days post inoculation. Values correspond to the mean diameter of 80 disease lesions from 20 apple fruits. The data is a representative of three independent experiments. Different letters on the column indicate significant differences between different treatments according to the least significant difference (LSD) test (*p* < 0.05).

Since c-di-GMP and YdaK regulate biocontrol efficacy against apple ring rot as mentioned above, we propose that c-di-GMP regulates biocontrol efficacy of PG12 through the potential receptor YdaK. To test this, the biocontrol assay was conducted to compare the biocontrol efficacy of PG12(pUBX) with the mutants Δ*dgcP*(pUBX-*pdeH*) and Δ*dgcPydaK*(pUBX-*pdeH*). The size of disease lesions on apples pre-treated with Δ*dgcPydaK*(pUBX-*pdeH*) was decreased to PG12(pUBX) level (the wild type level) compared to that with Δ*dgcP*(pUBX-*pdeH*; Figure 9), indicating that inhibition of biocontrol efficacy by low c-di-GMP level could be rescued through deletion of the *ydaK* gene and YdaK is necessary for biocontrol efficacy at low c-di-GMP level background. These results suggest that c-di-GMP regulates biocontrol efficacy of PG12 through the potential receptor YdaK.

### C-di-GMP regulates biofilm formation through YdaK in *B. velezensis*

**Figure 10:**
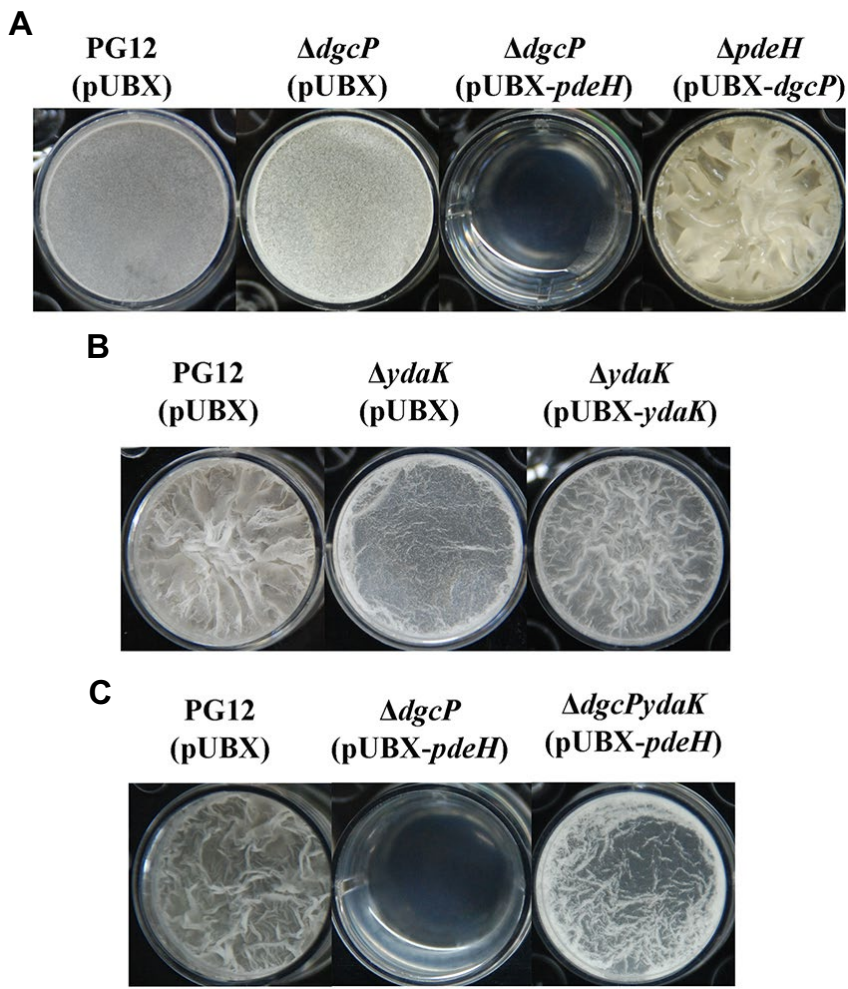
Biofilm formation of *B. velezensis* PG12(pUBX) and its derivatives. *B. velezensis* cells were grown in LB broth at 37°C overnight and then transferred to fresh LB broth to grow to mid-log phase (OD_600_ 0.8–1.0). 2 μl of the cultures mixed with 2 ml of MSgg broth supplemented with 1% xylose solution at the final concentration were transferred to the wells of 24-well microtiter plates. The plates were incubated at 28°C. Images were taken using a Nikon BM-7 digital camera after 48–72 h. **(A)** Microtiter plate assay of biofilm formation by *B. velezensis* PG12(pUBX) [PG12(pUBX)], Δ*dgcP*(pUBX), Δ*dgcP*(pUBX-*pdeH*), and Δ*pdeH*(pUBX-*dgcP*). **(B)** Microtiter plate assay of biofilm formation by *B. velezensis* PG12(pUBX) [PG12(pUBX)], Δ*ydaK*(pUBX) and its complemented strain Δ*ydaK*(pUBX-*ydaK*). **(C)** Microtiter plate assay of biofilm formation by *B. velezensis* PG12(pUBX) [PG12(pUBX)], Δ*dgcP*(pUBX-*pdeH*), and Δ*dgcPydaK*(pUBX-*pdeH*).

As shown above, c-di-GMP regulates biocontrol efficacy of PG12 through the potential receptor YdaK which is indispensable for an unknown EPS biosynthesis encoded by *ydaJKLMN* therefore biofilm formation in *B. subtilis*, but not through the PilZ domain protein MotI which is involved in c-di-GMP inhibition of motility in *B. subtilis* or the degenerated EAL protein YkuI which is implicated to control zinc homeostasis in *B. subtilis*. Therefore, we hypothesize that c-di-GMP regulates biocontrol efficacy through biofilm formation *via* YdaK in PG12. To test this hypothesis, we investigated whether c-di-GMP would regulate biofilm formation *via* YdaK. As expected, deletion of *dgcP* did not result in significant change in biofilm formation probably due to no change in c-di-GMP concentration in Δ*dgcP*(pUBX) compared to the wild-type PG12(pUBX) (Figures 2, 10A). The low c-di-GMP level strain Δ*dgcP*(pUBX-*pdeH*) showed less biofilm formation while the higher c-di-GMP level strain Δ*pdeH*(pUBX-*dgcP*) showed stronger biofilm formation compared to PG12(pUBX) (Figure 10A). These results indicate that c-di-GMP positively regulates biofilm formation in PG12. Furthermore, deletion of *ydaK* led to reducing biofilm formation and complementation of Δ*ydaK* with pUBX-*ydaK* restored biofilm formation to the wild-type level (Figure 10B), suggesting that YdaK positively regulates biofilm formation in PG12. Importantly, deletion of *ydaK* restored the reduced biofilm formation caused by low c-di-GMP concentration to the wild-type level (Figure 10C), suggesting that c-di-GMP regulates biofilm formation through YdaK.

### C-di-GMP regulates bacterial colonization on apple fruits through YdaK in *B. velezensis*

**Figure 11:**
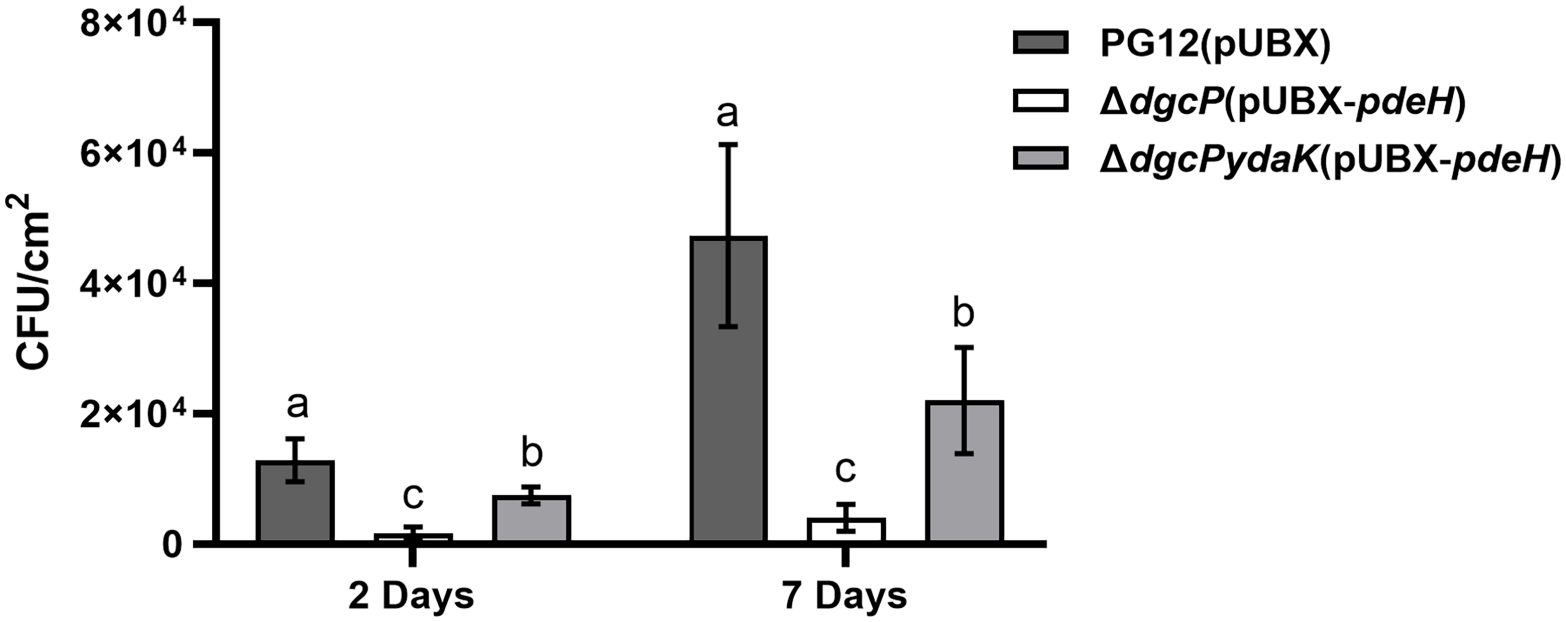
Bacterial population re-isolated from the surface of apple fruits treated with *B. velezensis* PG12(pUBX), Δ*dgcP*(pUBX-*pdeH*), or Δ*dgcPydaK*(pUBX-*pdeH*). Bacteria were incubated for 48 h in LB medium supplemented with 1% (w/v) xylose at the final concentration. Fuji apple fruits were soaked in the culture with 2.5 × 10^8^ cells/ml of *B. velezensis* PG12(pUBX) [PG12(pUBX)], Δ*dgcP*(pUBX-*pdeH*), or Δ*dgcPydaK*(pUBX-*pdeH*). Bacteria were isolated from the surface of the treated apple fruits at 2 and 7 days post inoculation, respectively. Values correspond to the mean bacterial population from three apple fruits. The data is a representative of two independent experiments. Different letters on the column indicate significant differences between different treatments according to the least significant difference (LSD) test (*p* < 0.05).

Since the ability to form biofilm is related to bacterial colonization efficiency on plants, we detected the bacterial colonization on apple fruits of PG12(pUBX) and its derivatives to determine whether c-di-GMP regulates colonization of PG12 on apple fruits or not. The data showed that significantly fewer bacteria were isolated from the surface of apple fruit treated with Δ*dgcP*(pUBX-*pdeH*) compared with PG12(pUBX) at 2 and 7 days post inoculation (Figure 11), suggesting less bacterial colonization in the low c-di-GMP background strain. No bacteria were isolated from the surface of apple fruits treated with LB broth. These results indicate that c-di-GMP positively regulates the colonization of PG12 on apple fruits. Moreover, deletion of *ydaK* restored the reduced colonization caused by low c-di-GMP level to the wild-type level (Figure 11), suggesting that c-di-GMP regulates bacterial colonization through YdaK. Based on the results above, we conclude that low c-di-GMP level in bacteria reduces biofilm formation and subsequently colonization on apple fruits, thereby reducing its biocontrol activity through the potential c-di-GMP receptor YdaK.

### Ydak does not affect the antagonistic activity of *B. velezensis* against *Botryosphaeria dothidea*

Since YdaK affects biocontrol efficacy of PG12 against apple ring rot disease and PG12 showed conspicuous antifungal activity against *B. dothidea*, the impact of YdaK on antagonistic activity of PG12 against *B. dothidea* was also investigated to determine whether YdaK regulates antagonistic activity and further biocontrol activity of PG12 against apple ring rot disease. The dual culture assay showed that there was no significant difference in the inhibition zone no matter between PG12(pUBX-*pdeH*) and Δ*ydaK*(pUBX-*pdeH*) or between Δ*dgcP* (pUBX-*pdeH*) and Δ*dgcPydaK*(pUBX-*pdeH*) (Figure 4), indicating that YdaK does not affect the antagonistic activity of PG12 against *B. dothidea in vitro*. As expected, deletion of either *motI* or *ykuI* also does not result in significant change in the antagonistic activity of PG12 against *B. dothidea* (Figure 4), indicating that YkuI and MotI also do not affect the antagonistic activity of PG12 against *B. dothidea in vitro*. Combining the results shown above that c-di-GMP did not affect the antagonistic activity of PG12 against *B. dothidea*, we conclude that c-di-GMP signaling pathway in PG12 regulates biocontrol efficacy against apple ring rot disease not through the direct inhibition of the pathogen.

*In toto*, we provide evidence that c-di-GMP regulates biofilm formation and subsequently colonization on apple fruits, thereby impacting the biocontrol efficacy of PG12 against apple ring rot disease *via* the essential potential c-di-GMP receptor YdaK. C-di-GMP signaling pathway does not regulate the antagonistic activity of PG12 against *B. dothidea*.

## Discussion

**Figure 12:**
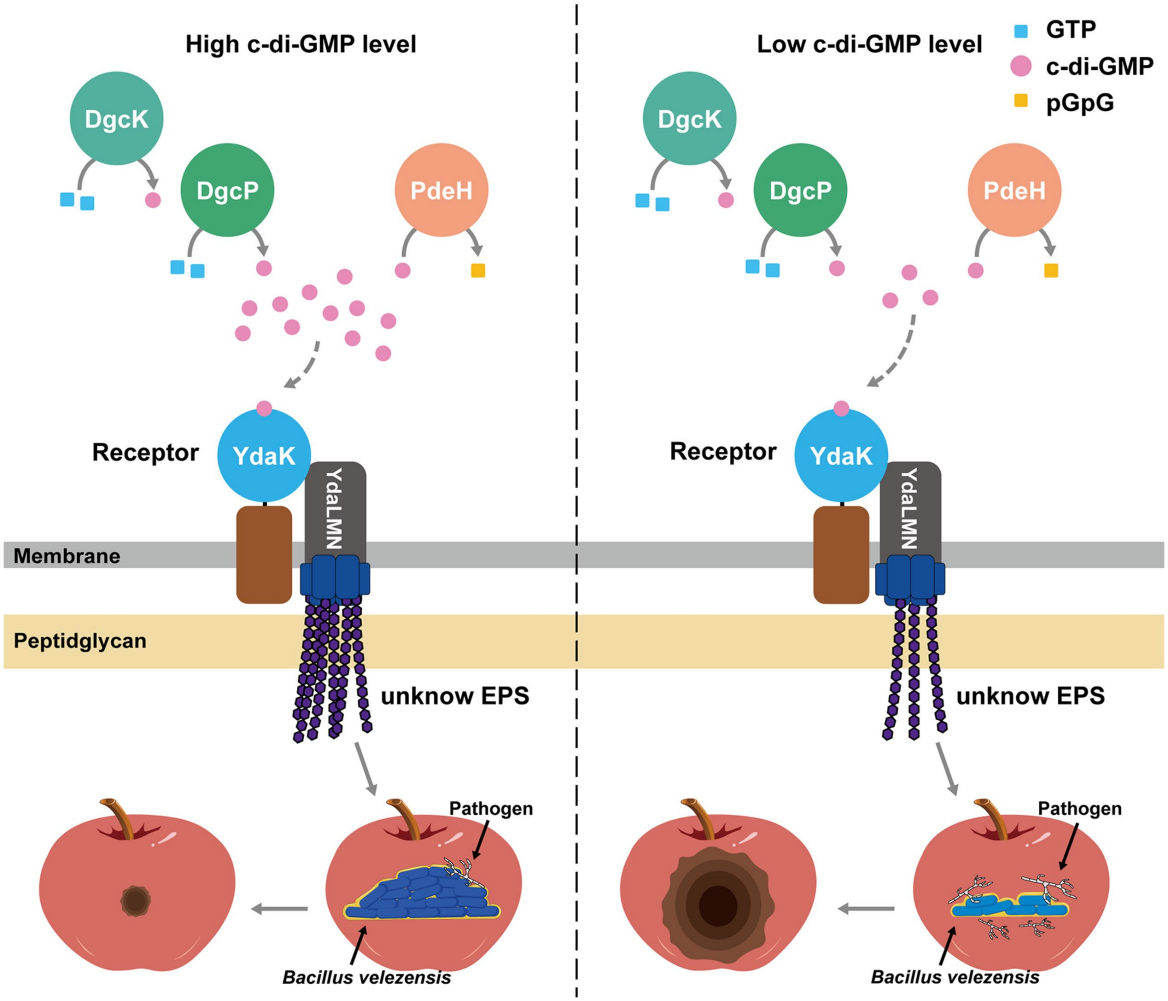
Model of c-di-GMP-dependent regulation of biocontrol efficacy *via* YdaK in *B. velezensis*. Two diguanylate cyclases (DGCs; DgcK, DgcP) synthesize c-di-GMP from GTP. The sole phosphodiesterases (PDE) PdeH degrades c-di-GMP into pGpG. The formation of the complex between the c-di-GMP receptor YdaK and the unknown EPS-synthesizing proteins YdaL, YdaM, and YdaN is essential for the catalytic activity of the latter proteins and for the synthesis of extracellular polysaccharides (EPS). Upon perception of an unknown extracellular signal, c-di-GMP is synthesized and binds to YdaK. C-di-GMP binding to the I-site motif of YdaK enables synthesis and export of the unknown EPS. When the c-di-GMP level is high in the cells, more EPS is synthesized, and strong biofilm is formed, enabling more bacteria colonizing on the apple fruits, thereby enhancing its biocontrol activity. When the c-di-GMP level is low in the cells, less EPS were synthesized, and less biofilm is formed, enabling less bacteria colonize on the apple fruits, thereby reducing its biocontrol activity. Solid and dashed grey arrows indicate proved and not proved regulation in *B. velezensis*, respectively.

In this study, we investigated the role of c-di-GMP in biocontrol activity of *B. velezensis* against apple ring rot disease and determined its regulatory pathway. We generated diverse combinations of overexpression and deletion mutants for c-di-GMP signaling genes in *B. velezensis* PG12 and studied their effects on biocontrol activity against apple ring rot disease. Our results showed that artificial modulation of c-di-GMP level leads to significantly change of biocontrol efficacy of PG12. In addition, c-di-GMP regulates biofilm formation and bacterial colonization on apple fruits. Evidence also showed that YdaK could rescue the reduced biofilm formation, bacterial colonization on apple fruits and biocontrol efficacy caused by low level of c-di-GMP. However, neither c-di-GMP nor YdaK regulates the antagonistic activity of PG12 against *B. dothidea*. Collectively, our findings support the idea that c-di-GMP plays an important role in regulating biocontrol efficacy of *B. velezensis* against apple ring rot disease through its regulation on biofilm formation and thus colonization on apple fruits *via* the essential potential c-di-GMP receptor YdaK (Figure 12).

Unlike many other bacteria, none of our single mutants of DGCs had a conspicuous effect on biocontrol efficacy of *B. velezensis* PG12 against apple ring rot disease. This observation is in agreement with what has been seen in *B. subtilis* and *B. anthracis* for which single or multiple mutations of genes encoding DGCs do not have a detectable phenotype (Chen et al., 2012; Gao et al., 2013; Hermanas et al., 2021). It is likely due to functional redundancy among the encoded proteins. We have not, however, constructed a c-di-GMP null mutant. Biofilm formation is not affected by no matter the deletion of all diguanylate cyclases or overexpression of diguanylate cyclase in *B. subtilis* (Chen et al., 2012; Gao et al., 2013). However, overexpression of diguanylate cyclase resulted in significant change of biofilm formation in *B. velezensis* (Figure 10A). Therefore, we speculate that the c-di-GMP level in wild-type *B. velezensis* is higher than that in *B. subtilis*, and there are thresholds of c-di-GMP concentrations to cause the significant change of biofilm formation. In addition, *B. velezensis* shares the same DgcK, DgcP, and PdeH with *B. subtilis*, but does not have the GGDEF-EAL domain tandem protein DgcW which possesses diguanylate cyclase activity in *B. subtilis* (Gao et al., 2013; Yang et al., 2018). Therefore, we speculate that different mechanisms of c-di-GMP metabolism in *B. velezensis* compared to *B. subtilis* causing different c-di-GMP concentrations in the wild-type strains.

The c-di-GMP signaling has been widely studied in the pathogenic bacteria, such as *E. coli*, *Vibrio fischeri*, *Erwinia amylovora*, *Xanthomonas campestris*, *X. oryzae* pv. *oryzae* (Yang et al., 2012). For beneficial bacteria, where the physiological role of c-di-GMP is not as well-characterized as in pathogenic bacteria, c-di-GMP was demonstrated to influence, for example, the interactions between the rhizobium *Sinorhizobium meliloti* and its host plants to establish efficient symbiosis (Wang et al., 2010), the production of antibiotic production in *Streptomyces coelicolor* and *L. enzymogenes* (Den Hengst et al., 2010; Xu et al., 2018). Since production of antimicrobials is a crucial mechanism for beneficial bacteria to control plant pathogens, it will be interesting to find out whether c-di-GMP also regulate antifungal activity in *B. velezensis*. However, our results revealed that c-di-GMP did not affect the antagonistic activity of PG12 to the pathogenic fungal *B. dothidea* both *in vitro* and *in vivo*, indicating that c-di-GMP does not regulate antimicrobials production active to *B. dothidea* in *B. velezensis* under our conditions. Significantly, our work revealed that c-di-GMP influences biocontrol activity of *B. velezensis* against apple ring rot disease. Our findings significantly extend previous knowledge on c-di-GMP functions to include biocontrol activity.

Bacteria in nature are exposed to a constantly changing environment and their ability to sense and react to these changes is important for their survival. Numerous studies imply that c-di-GMP is a crucial signaling molecule to transduce the external stimuli into a cellular response and helps the bacteria adapt to environmental changes (Monds et al., 2007; Plate and Marletta, 2012; Townsley and Yildiz, 2015). For example, in *Yersinia pestis*, environmental signals regulate biofilm formation through regulation of the c-di-GMP level, and DGCs and PDEs differentially respond to environmental changes (Ren et al., 2016). Our results showed that significant change of biofilm formation, subsequently colonization and therefore biocontrol efficacy was observed only when diguanylate cyclase or phosphodiesterase encoding gene was artificially overexpressed in *B. velezensis* PG12. We speculate that the synthesis of c-di-GMP is one strategy *B. velezensis* employs to sense alterations in their environment and rapidly adjust to those changes. The environmental factors that affect the level of c-di-GMP will be interesting to be identified in future studies.

In *B. subtilis*, c-di-GMP activates the production of an unknown EPS synthesized by YdaK-N and thus potential biofilm formation *via* the receptor YdaK (Bedrunka and Graumann, 2017a,b). However, the function of EPS production by YdaLMN is unclear. Homologs of YdaK was also found in *B. velezensis*. Our data indicate that YdaK serves as a c-di-GMP receptor to modulate EPS production and thus biofilm in *B. velezensis* (Figure 12), which is consistent with what was previously reported in *B. subtilis*. We have not, however, directly demonstrated the binding affinity of c-di-GMP and YdaK in *B. velezensis* PG12. In addition, biofilm formation is important for bacterial colonization and thus plant protection. Increasing studies showed that c-di-GMP is essential for host colonization including *Azoarcus* sp. CIB, *E. amylovora* and *V. fischeri* (Fernandez-Llamosas et al., 2021; Isenberg et al., 2022; Kharadi et al., 2022). Our data indicate that c-di-GMP affects bacterial colonization on apple fruits *via* the potential receptor YdaK (Figure 12), which is consistent with that previously reported in *Azoarcus* sp. CIB, *E. amylovora* and *V. fischeri*. Since both c-di-GMP and YdaK do not regulate antimicrobials production active to *B. dothidea* in *B. velezensis* under our conditions, we provided evidence that c-di-GMP positively regulates biofilm formation, subsequently colonization, and therefore biocontrol activity *via* the potential receptor YdaK. Our study provides an evidence suggesting a causal role for biofilm formation produced by the unknown EPS in colonization and subsequently biocontrol efficacy.

## Data availability statement

The original contributions presented in the study are included in the article/Supplementary material, further inquiries can be directed to the corresponding author.

## Author contributions

HG constructed stains and performed the biocontrol assay, antagonistic assay and biofilm assay, and the statistical analysis. WJ performed the biocontrol assay, the assay of bacterial colonization on apple fruits, and drew the model. YY and YZ constructed mutants. XC and WL performed the biocontrol assay. PY performed the assay of bacterial colonization on apple fruits. ZW and QW contributed to analysis the data. YL designed the study, analysis the data, and wrote the manuscript. All authors contributed to the article and approved the submitted version.

## Funding

This project was supported by grants from National Natural Science Foundation of China (nos. 31672074, 31972982 and 32272613), the Beijing Municipal Natural Science Foundation (no. 6172018), and Chinese Universities Scientific Fund (nos. 2021TC073 and 2020TC044).

## Conflict of interest

The authors declare that the research was conducted in the absence of any commercial or financial relationships that could be construed as a potential conflict of interest.

## Publisher’s note

All claims expressed in this article are solely those of the authors and do not necessarily represent those of their affiliated organizations, or those of the publisher, the editors and the reviewers. Any product that may be evaluated in this article, or claim that may be made by its manufacturer, is not guaranteed or endorsed by the publisher.

## Supplementary material

The Supplementary material for this article can be found online at: https://www.frontiersin.org/articles/10.3389/fmicb.2022.1034168/full#supplementary-material
