# Supplementary material for: Cyclic di-GMP regulates bacterial colonization and further biocontrol efficacy of Bacillus velezensis against apple ring rot disease via its potential receptor YdaK
Source: Front Microbiol. 2022 Dec 16;13:1034168. doi: 10.3389/fmicb.2022.1034168 (PMC9800504; doi:10.3389/fmicb.2022.1034168)
Supplement: Supplementary file 1 [file Data_Sheet_1.docx]

Supplementary Material

# Supplementary Figures and Tables

## Supplementary Figure
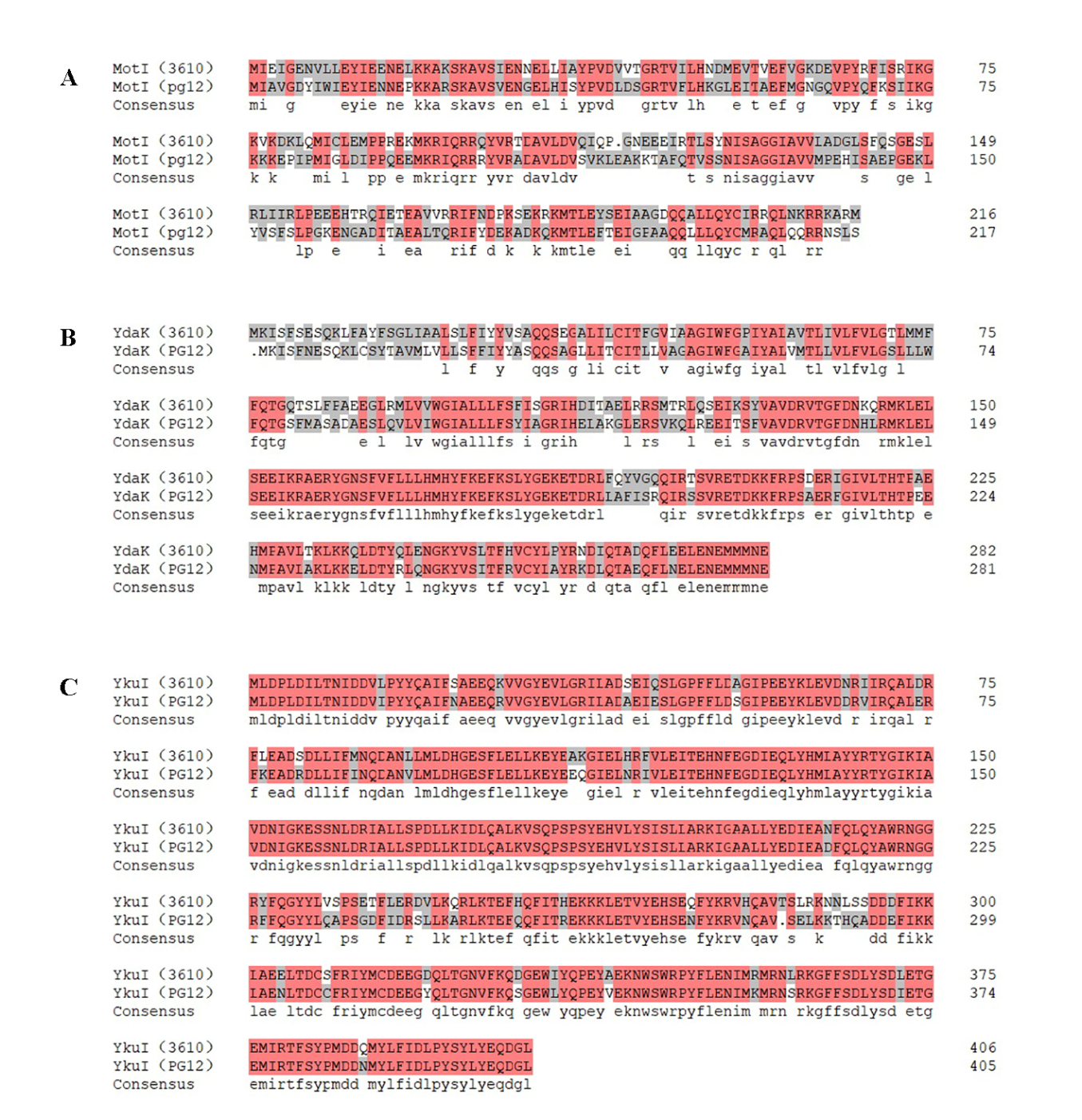


**Supplementary Figure S1. Alignment of the amino acid sequences of MotI (A), Ydak (B) and YkuI (C) in *B. velezensis* PG12 and *B. subtilis* 3610.** The number of amino acids (aa) in each protein is shown. The consensus sequence was highlighted in red. The sequence alignment was created using DNAMAN 9.0.1.

## Supplementary Table

# Supplementary Table S1 Primers used in the study.

| **Primer name** | **Primer sequence^a^** | **Amplicon** |
| --- | --- | --- |
| *motI*-Up-F | CCCaagcttTTTCTGAAGACAGTACCAGCCCTAC | *motI* deletion |
| *motI*-Up-R | CGGactagtCAGCTATCATGGCCTTCACTCT |  |
| *motI*-Dn-F | CGGactagtCAGCTATCATGGCCTTCACTCT |  |
| *motI*-Dn-R | CCGgaattcTCGGCATCAGGCAGCAC |  |
| *motI*-in-1682F | AAATGTTGAAGTGCGGGAGAC | *motI* deletion confirmation |
| *motI*-in-3178R | CTTCTGTCACATCTTCTCCGTTTA |  |
| *motI*-out-957F | CGCAGCGTTCAGCATCTCGT |  |
| *motI*-out-3807R | CCTCCTTGGTCCAAACCTACAC |  |
| *ydaK* -Up-F | AActgcagAAACGGGCTCGTGTCGTGG | *ydaK* deletion |
| *ydaK* -Up-R | CGGactagtTCATTGAATGATATTTTCATTAAAC |  |
| *ydaK* -Dn-F | CGGggtaccGGAAAACGAGATGATGATGAATGAA |  |
| *ydaK* -Dn-R | CCGgaattcGCCGCAAGTAAAGGAGGGTG |  |
| *ydaK*-in-1463F | ACCGTGACCTTGTCCTATGTTGA | *ydaK* deletion confirmation |
| *ydaK*-in-3054R | TGTGATGTCTCCGTCGCTTTT |  |
| *ydaK*-out-922F | AGGAGGATGGACTGTGAAGCAA |  |
| *ydaK*-out-4245R | GCCGAGATACGGATGGTAGAAA |  |
| pUBX-SL-F | ATCGAACAATTGAATCTCTAGAGGATCTTCCTTCAGGTT | pUBX*-ydaK* construction, *ydaK* deletion complementation |
| pUBX-SL-R | TGTTTTCTGACTCATATCGAACATTTCCCCCTTTGATTTTTAGAT |  |
| *ydaK*-SL-F | AATGTTCGATATGAGTCAGAAAACAATGAAAATATCATTCAATGAATCAC |  |
| *ydaK*-SL-R | TCCTCTAGAGATTCAATTGTTCGATTTAGAGTTCATTCATCATCATCTC |  |
| pUBX-F | GTTGATGGATAAACTTGTTCACT | pUBX*-ydaK* confirmation |
| pUBX-R | CGATTCCAGAAGTTTCTCAGAGT |  |

# ^a^ The restriction sites are underlined lowercase.
